# A retrospective comparative analysis of factors affecting the decision and outcome of initial intravenous immunoglobulin alone or intravenous immunoglobulin plus methylprednisolone use in children with the multisystem inflammatory syndrome

**DOI:** 10.1186/s12969-022-00726-2

**Published:** 2022-08-20

**Authors:** İlker Devrim, Elif Böncüoğlu, Elif Kıymet, Şahika Şahinkaya, Miray Yılmaz Çelebi, Ela Cem, Mine Düzgöl, Kamile Ötiken Arıkan, Aybüke Akaslan Kara, Dorukhan Besin, Gamze Vuran, Pınar Seven, Timur Meşe, Hasan Ağın, Nuri Bayram

**Affiliations:** 1 Department of Pediatric Infectious Diseases, Dr. Behçet Uz Child Disease and Pediatric Surgery Training and Research Hospital, Izmir, Turkey; 2 Department of Pediatrics, Dr. Behçet Uz Child Disease and Pediatric Surgery Training and Research Hospital, Izmir, Turkey; 3 Department of Pediatric Cardiology, Dr. Behçet Uz Child Disease and Pediatric Surgery Training and Research Hospital, Izmir, Turkey; 4 Department of Pediatric Intensive Care, Dr. Behçet Uz Child Disease and Pediatric Surgery Training and Research Hospital, Izmir, Turkey

**Keywords:** Intravenous immunoglobulin, Methylprednisolone, Multisystem inflammatory syndrome, COVID-19

## Abstract

**Background:**

For children with the multisystem inflammatory syndrome(MIS-C), intravenous immunoglobulins (IVIG) with or without methylprednisolone are the most effective treatment. In this study, IVIG combined with methylprednisolone was compared to IVIG used alone in children with MIS-C.

**Methods:**

This retrospective cohort study was carried out between April 1, 2020, and November 1, 2021. This study covered all children with MIS-C. According to whether they received IVIG alone or IVIG with methylprednisolone as an initial treatment for MIS-C, the patients were split into two groups. The IVIG dosage for the patients in group I was 2 gr/kg, whereas the IVIG dosage for the patients in group II was 2 gr/kg + 2 mg/kg/day of methylprednisolone. These two groups were contrasted in terms of the frequency of fever, length of hospital stay, and admission to the pediatric intensive care unit.

**Results:**

The study comprised 91 patients who were diagnosed with MIS-C and were under the age of 18. 42 (46.2%) of these patients were in the IVIG alone group (group I), and 49 (53.8%) were in the IVIG + methylprednisolone group (group II). Patients in group II had a severe MIS-C ratio of 36.7%, which was substantially greater than the rate of severe MIS-C patients in group I (9.5%) (p 0.01). When compared to group I (9.5%), the rate of hypotension was considerably higher in group II (30.6%) (*p* = 0.014). Additionally, patients in group II had considerably higher mean serum levels of C-reactive protein. The incidence of fever recurrence was 26.5% in group II and 33.3% in group I, however the difference was not statistically significant (*p* > 0.05).

**Conclusions:**

The choice of treatment for patients with MIS-C should be based on an individual evaluation. In MIS-C children with hypotension and/or with an indication for a pediatric intensive care unit, a combination of IVIG and methylprednisolone may be administered. For the treatment modalities of children with MIS-C, however, randomized double-blind studies are necessary.

## Introduction

Children appeared to have a milder course of COVID-19(Coronavirus Disease 2019) infections than adults, according to studies from the early days of the pandemic [1]. However, a group of children with the signs and test results of a hyperinflammatory course linked to COVID-19 infections have been observed since April 2020 [2–5]. These children resembled toxic shock syndrome, atypical Kawasaki disease, and Kawasaki disease shock syndrome before being diagnosed with multisystem inflammatory syndrome in children (MIS-C) [6–8]. The World Health Organization (WHO) and the Centers for Disease Control and Prevention (CDC) defined this immune system’s post-infectious inflammatory response and published their diagnostic criteria [9, 10]. While it was typically estimated to be about 1% in high-income nations, the mortality rate of MIS-C has been shown to be as high as 9% in other reports, demonstrating its significance for public health [11–14].

Most of the MIS-C therapy options are inspired by Kawasaki illness and the new knowledge gained from treating individuals with COVID-19 who have been diagnosed with an exacerbating condition known as a cytokine storm. Currently, the most popular form of treatment is intravenous immunoglobulin (IVIG), either alone or in conjunction with corticosteroids [15–19]. Other medications or therapeutic approaches, such as plasmapheresis or interleukin 1 inhibitors, were used in patients that were either non-responsive or severe [6, 20, 21]. The research primarily focusing on the treatment options for children with MIS-C are few during the first year of the COVID-19 epidemic, and the majority of the therapy recommendations are drawn from institutional procedures.

The purpose of this study was to assess the available therapy options for kids with MIS-C and to compare the effectiveness and indications for IVIG combined with methylprednisolone against IVIG alone in kids with MIS-C.

## Methods

### Patients and settings

This retrospective cohort study was carried out at the Dr. Behçet Uz Children’s Hospital of the Health Sciences Faculty of Medicine between April 1, 2020, and November 1, 2021. In Turkey’s Aegean Region, the hospital serves as a pediatric patients’ referral facility. The study included all children identified as having MIS-C in accordance with CDC guidelines [9]. The criteria for a conclusive diagnosis included epidemiological association to COVID-19 in addition to prolonged fever, elevated inflammatory biomarkers, indications of multi-organ involvement, and the exclusion of any other diagnosis [9]. Through nasopharyngeal real-time reverse transcription polymerase chain reaction analysis and/or SARS-CoV-2 antibody testing, all patients had to demonstrate that they had been exposed to the severe acute respiratory syndrome coronavirus type 2 (SARS-CoV-2). Four weeks prior to the development of clinical symptoms, exposure to a suspected or confirmed COVID-19 case was also noted. Several molecular and microbiological diagnostic tests, such as multiplex PCR tests for common respiratory pathogens, rapid antigen tests for influenza, serological tests for the Epstein-Barr virus, conventional culture tests, such as blood and throat cultures in addition to peripheral smears, ultrasonography, etc., were used to rule out other diagnoses.

According to the measurements of infrared thermometers, fever was defined as a temperature of 38 °C or higher [22]. The patients who had fever after the end of IVIG were noted and the time of re-occurrence of fever after the end of the IVIG therapy was recorded. Through the use of the electronic medical record system and patient files, demographic information, symptoms, medical history, and distinguishing characteristics of the patients were gathered.

According to whether they received IVIG alone or IVIG with methylprednisolone as an initial treatment for MIS-C, the patients were split into two groups. The IVIG dosage for the patients in group I was 2 gr/kg, whereas the IVIG dosage for the patients in group II was 2 gr/kg + 2 mg/kg/day of methylprednisolone.

SPSS Statistical Software was used to conduct the statistical analysis (version 22; SPSS, Chicago, IL, USA). We compared categorical variables using Fisher’s exact and Pearson’s 2 tests. The Mann–Whitney U test or the t test were used to compare numerical variables (depending on whether they show normal distribution or not). Categorical variables were given as frequencies and percentages, whereas continuous variables were shown as means and standard deviation.

The Institutional Review Board of the Dr. Behcet Uz Children’s Training and Research Hospital gave ethics approval for this study.

### Definitions and treatments

According to the measurements of infrared thermometers, fever was defined as a temperature of 38 °C or higher [22]. The patients who had fever after the end of IVIG were noted and the time of re-occurrence of fever after the end of the IVIG therapy was recorded. Through the use of the electronic medical record system and patient files, demographic information, symptoms, medical history, and distinguishing characteristics of the patients were gathered.

According to whether they received IVIG alone or IVIG with methylprednisolone as an initial treatment for MIS-C, the patients were split into two groups. The IVIG dosage for the patients in group I was 2 gr/kg, whereas the IVIG dosage for the patients in group II was 2 gr/kg + 2 mg/kg/day of methylprednisolone.

### Statistics

SPSS Statistical Software was used to conduct the statistical analysis (version 22; SPSS, Chicago, IL, USA). We compared categorical variables using Fisher’s exact and Pearson’s 2 tests. The Mann–Whitney U test or the t test were used to compare numerical variables (depending on whether they show normal distribution or not). Categorical variables were given as frequencies and percentages, whereas continuous variables were shown as means and standard deviation.

The Institutional Review Board of the Dr. Behcet Uz Children’s Training and Research Hospital gave ethics approval for this study.

## Results

This study included 91 individuals with MIS-C in total. There were 30 (33%) female patients and 61 (67.0%) male patients. The average age of the patients was six and a half years (range 5 months to 17 years). Eighty-four (92.3%) of the 91 patients were children were previously healthy.

### Epidemiological and clinical information

SARS-CoV-2 real-time PCR findings were positive in 4 (4.4%) of the 91 individuals with MIS-C. 42.9% of MIS-C cases (*n* = 39) had a history of contact with a COVID-19 case. Seventy (76.9%) patients had positive for COVID-19 immunoglobulin G and seven patients (7.7%) had positive for COVID-19 immunoglobulin M.

Twenty percent of the patients (*n* = 19) had hypotension, while twenty percent of the patients (*n* = 20) had tachycardia. Eight patients (8.8%) experienced breathing problems. Table 1 lists the patients’ symptoms in brief. Systolic dysfunction, defined as an ejection fraction (EF) below 50%, was seen in 10 (11.0%) of the 91 patients, while coronary artery involvement was seen in 5 (5.5%) of the patients. Pericardial effusion was experienced by one patient (1.1%). Thirty-three (36.2%) of the patients had mitral valve regurgitation (Table 1).

**Table 1:** The symptoms and features of the children with multisystem inflammatory syndrome in children

Presenting symptoms	Group I (IVIG alone) (%)	Group II (IVIG plus steroid) (%)	Total Number (%)	*P* value
Fever	42(100)	49 (100)	91(100)	
Cough	8(19.0%)	2 (4.1%)	10 (11.0)	0.04
Abdominal pain	13(31.0)	19(38.8)	32(35.2)	> 0.05
Chest pain	1(2.4)	2(4.1)	3(3.3)	> 0.05
Vomitting	11(26.2)	22(44.9)	33(36.3)	> 0.05
Diarrhea	10(23.8)	17(34.7)	27(29.7)	> 0.05
Rash	11(26.2)	17(34.7)	28(30.8)	> 0.05
Edema of the extremities	6(14.3)	8(16.3)	14 (15.4)	> 0.05
Periungal desquamation	5(11.9)	2(4.1)	7(7.7)	NA
Conjuctivitis	12 (28.6)	17 (34.7)	29(31.9)	> 0.05
Headache	1 (2.4)	4(8.3)	5(5.5)	NA
Cervical Lympadenopathy	4(9.5)	4(8.2)	8(8.8)	NA
**Clinical evaluation**				
Hypotension	4(9.5)	15 (30.6)	19(20.9)	0.014
Tachycardia	10 (23.8)	10 (20.4)	20(22.0)	> 0.05
Tachypnea	4(9.5%)	4 (8.2)	8(8.8)	> 0.05
Echo findings				
**Systolic dysfunction**	3(7.1)	7(14.3)	10(11)	> 0.05
Coronary artery involvement	4(9.5)	1(2.0)	5(5.5)	NA
Pericardial effusion	-	1(2.0)	1(1.1)	NA
Mitral valve regurgitation	15(35.7)	18(36.7)	33(36.2)	> 0.05
**Severity score**				0.01*
Mild	28(66.7)	23 (46.9)	51(56.0)	
Moderate	10( 23.8)	8 (16.3)	18 (19.8)	
Severe	4(9.5)	18(36.7)	22(24.2)	
Admission to PICU	6 (14.3)	16(32.7)	22(24.2)	0.032

^a^The ratio of severe MIS-C patients in the group II was 36.7% (*n* = 18) and significantly higher compared to the rate of severe MIS-C patients in the group I (*n* = 4, 9.5%) (*p* = 0.01)

*NA* Not applicable, *PICU* Pediatric Intensive Care Unit

### Treatment modalities of the MIS-C

The initial treatment was intravenous immunoglobulin alone at 42 patients (46.2%) and intravenous immunoglobulin plus methylprednisolone was administered at 49 patients (53.8%). Eighteen patients (19.8%) required inotropic agents and 14 patients (15.4%) required respiratory support including high-flow nasal cannula. Of 91 patients, 22 (24.2%) were followed up in the pediatric intensive care unit (PICU). During follow-up, no mortality was observed in our study cohort.

### What effect the decision for an initial steroid to IVIG at MIS-C duration of fever and time to start IVIG

The median age of group II patients was significantly older than that of group I patients (96 months, 5 to 168 months vs 51 months, 16 to 204; *p* < 0.001). The proportion of severe MIS-C patients in group II was 36.7% (*n* = 18), which was considerably greater than the proportion of severe MIS-C patients in group I (*n* = 4, 9.5%) (*p* = 0.01). In group II, the rates of moderate and mild MIS-C were 16.3% (*n* = 8) and 46.9% (*n* = 23), respectively. In group I, the rates of moderate and mild MIS-C were 66.7% (*n* = 28) and 23.8% (*n* = 10), respectively. The admission rate to the PICU (Pediatric Intensive Care Unit) was 34.7% in group II and 14.3% in group I, with the difference being substantially greater in group II (*p* = 0.026). The incidence of hypotension was substantially higher in group II (30.6%; *n* = 15) compared to group I (9.5%; *n* = 4) (*p* = 0.014). There was no significant difference between the two groups in terms of EF (*p* > 0.05).

In group II, the platelet and lymphocyte counts were considerably lower than in group I (*p* = 0.005 and *p* = 0.0119, respectively) (Table 2). The mean serum levels of C-reactive protein (CRP) were considerably higher in group II, although there were no significant differences between the two groups for other laboratory variables (*p* > 0.05).

**Table 2:** The comparison of laboratory parameters between the groups with IVIG versus IVIG plus methylprednisolone

	IVIG alone (*n* = 42)	IVIG plus methylprednisolone (*n* = 49)	*p* value
WBC ( cells/µL)	11,506 (2130–23,800)	10,445 (1050–26,170)	> 0.05
ANC ( cells/µL)	7737 (750–16,290)	8217 (2240–23,430)	> 0.05
ALC ( cells/µL)	2635 (510–9060)	1685 (270–7600)	0.01
PLT ( cells/µL)	242,238 (84,000–546,000)	188,204 (61,000–419,000)	0.005
Fibrinogen (mg/dL)	616 (350–1496)	630 (320–1264)	> 0.05
D-dimer (ng/mL)	957 (150–3883)	1240 (150–9924)	> 0.05
CRP (mg/dL)	11.6 (0.00–32.3)	15.4 (0.00- 35.7)	0.043
ESR (mm/h)	68.27 (18–142)	72.14 (5–133)	> 0.05
Ferritin (µg/L)	558.66 (54.7–2572)	778.99 (77.24–5546)	> 0.05

*ALC* Absolute lymphocyte count, *ANC* Absolute neutrophil count, *CRP* C-reactive protein, *ESR* Erythrocyte sedimentation rate, *PLT* Platelet count, *WBC* White blood cell count

### The prognosis and the treatment of choice

The recurrence of fever was 26.5% (*n* = 13) in group II and 33.3% (*n* = 14) in group I, although there was no statistically significant difference (*p* < 0.05). The rate of respiratory support was 14.3% (*n* = 7) in group II and 9.5% (*n* = 4) in group I; however, there was no statistically significant difference between the groups (*p* > 0.05). The mean hospitalization duration was 9.9 ± 1.2 days (2 to 55 days in the group I and 10.4 ± 0.6 days (4 to 20 days) in the group II and no significant difference was present between these two groups (*p* > 0.05).

## Discussion

In this study, we discussed our experience with MIS-C in children and the outcomes of patients treated with IVIG alone or in conjunction with corticosteroids. In 46.2% of 91 patients, intravenous immunoglobulin was supplied alone, whereas intravenous immunoglobulin plus steroid was administered in 53.8% of patients. Children admitted to the PICU and/or suffering from hypotension were more likely to receive a combination of corticosteroids and IVIG. We discovered no statistically significant difference between the effects of two treatment strategies on the length of hospital stay and recovery from fever when we compared their effects on the outcome.

According to systemic reviews conducted at the onset of the pandemic, IVIG was the most often employed treatment modality, with a rate of 76.4% among 662 patients, followed by corticosteroids and other medications, including corticosteroids, with a rate of 52.3% [23]. However, towards the end of a year of the COVID-19 pandemic, the combination of IVIG and steroids has been incorporated into the main treatment protocols [24]. A recent retrospective analysis involving 181 MIS-C patients that examined the response of IVIG alone and in conjunction with steroid treatment approaches revealed that the use of IVIG in combination with steroid treatment is associated with a more positive outcome [24]. While the rate of failure to respond in terms of fever was % in the IVIG and methylprednisolone group and 51% in the IVIG group, the failure rate was much higher in the IVIG group [23]. Similarly, in a different study, Belhadjer et al. found that IVIG + steroids was related with a quicker cardiac recovery in patients with MIS-C [15]. In the early stages of the COVID-19 pandemic, the United Kingdom recommendations advocated the use of IVIG alone [18], but more recent papers favored the first combination of IVIG and corticosteroids [15, 24]. In addition, according to a recent study by Ouldali et al., among the 72 patients with MIS-C in the IVIG-alone treatment group, corticosteroids were added to the treatment regimen for only 13 patients (18.1%) [24].

In patients with MIS-C, the decision to add a steroid to the IVIG upon admission is not governed by specific criteria. In two separate Turkish investigations, Ozsurekci et al. and Alkan et al. found that all patients received a high dose of IVIG and corticosteroids concurrently [25, 26]. Indications for initial IVIG and steroid combination were primarily related to the clinical severity of the patient, such as depressed EF, presence of hypotension, and/or respiratory insufficiencies, which were primarily included in the criteria for moderate and severe MIS-C diagnosis [19]. In our analysis, the risk of severe MIS-C and hypotension was much greater in the combination treatment, which likely resulted in more admissions to the PICU. Therefore, in the case of hypotension and severe MIS-C, physicians favored adding methylprednisolone to IVIG initially.

In this study, the outcome factors, such as the rate of hospitalization in the PICU, duration of fever, and length of stay in the hospital, do not differ between the steroid combination group and the control group, which is contrary to the findings of prior research [15, 24]. Regarding recovery fever, there was no significant difference between the IVIG and IVIG + steroid groups in our study. Additionally, the current study found no difference in hospitalization duration between the IVIG plus steroid group and the other groups, which may be attributable to selection bias. Since this was not a randomized controlled trial, physicians tended to combine methylprednisolone with intravenous immunoglobulin (IVIG) in critically ill patients who were anticipated to require prolonged hospitalization and rehabilitation periods. Moreover, it must be underlined that we did not detect any death during the research period, and that just one patient required immunomodulatory therapy, indicating the potential efficacy of existing treatment regimens for patients with MIS-C.

The design of this study has a number of limitations. First, because it was not a randomized controlled study, it is unable to control for probable confounding variables that led to bias. The decision to first add steroids to IVIG was mostly subjective; for instance, IVIG in combination with steroids was delivered to more critically ill patients, which influences outcome-related variables such as length of hospital stay. To counteract this bias, we attempted to establish criteria for the definitions of severe and moderate MIS-C [19]. Moreover, the sample size is too small to generalize our findings; yet, given the paucity of studies concentrating on the treatment of MIS-C, this investigation provides physicians with extra helpful information.

## Conclusions

The decision of whether to treat MIS-C patients with IVIG plus methylprednisolone or IVIG alone must be reviewed on an individual basis. IVIG in conjunction with methylprednisolone should be considered for patients with severe MIS-C and hypotension who have been admitted to the PICU. The treatment options for children with MIS-C still require randomized, double-blind research.

## Data Availability

The data and materials supporting the conclusions of the study are available from the corresponding author on reasonable request.

## Abbreviations

CDC: Centers for Disease Control and Prevention
COVID-19: Coronavirus disease 2019
CRP: C-reactive protein
EF: Ejection fraction
IVIG: Intravenous immunoglobulin
MIS-C: Multisystem inflammatory syndrome in children
SARS-CoV-2: Severe acute respiratory syndrome coronavirus type 2
PICU: Pediatric Intensive Care Unit
WHO: World Health Organization
